# The characteristics of premature infants with transient corneal haze

**DOI:** 10.1371/journal.pone.0195300

**Published:** 2018-03-29

**Authors:** Yu-Hung Lai, Hsiu-Lin Chen, San-Nan Yang, Shun-Jen Chang, Lea-Yea Chuang, Wen-Chuan Wu

**Affiliations:** 1 Department of Ophthalmology, Kaohsiung Medical University Hospital, Kaohsiung Medical University, Kaohsiung, Taiwan; 2 Department of Ophthalmology, School of Medicine, College of Medicine, Kaohsiung Medical University, Kaohsiung, Taiwan; 3 Graduate Institute of Medicine, College of Medicine, Kaohsiung Medical University, Kaohsiung, Taiwan; 4 Department of Pediatrics, Kaohsiung Medical University Hospital, Kaohsiung Medical University, Kaohsiung, Taiwan; 5 Department of Respiratory Therapy, College of Medicine, Kaohsiung Medical University, Kaohsiung, Taiwan; 6 Department of Pediatrics, E-DA Hospital and School of Medicine, College of Medicine, I-Shou University, Kaohsiung, Taiwan; 7 Department of Kinesiology, Health, and Leisure Studies, College of Humanities and Social Sciences, National University of Kaohsiung, Kaohsiung, Taiwan; 8 Department of Biochemistry, College of Medicine, Kaohsiung Medical University, Kaohsiung, Taiwan; Children's Hospital Boston, UNITED STATES

## Abstract

**Background:**

The etiology of transient corneal haze in premature infants is not known and how it relates to clinical outcomes in premature infants is not clear.

**Objectives:**

To study associated factors of transient corneal haze in premature infants.

**Methods:**

We performed a retrospective study of 261 premature infants from retinopathy of prematurity (ROP) screening in the neonatal intensive care unit at a tertiary referral hospital. Characteristics of premature infants with and without corneal haze were analyzed by correlation tests, Chi-square tests, and logistic regressions were used for statistical analyses. Associations between corneal haze and birth weight (BW), gestational age at birth (GA), central corneal thickness, intraocular pressure, and other systemic and ophthalmic data were evaluated.

**Results:**

The incidence of corneal haze was 13.4%. Lower BW, lower GA, packed red blood cells (RBC) transfusion, more days on oxygen, older maternal age, bronchopulmonary disease, and stage 3 ROP are associated with corneal haze. The severity of corneal haze decreased with infants’ postmenstrual age. Multivariate logistic regression analyses revealed that BW and maternal age are the most important predictors of corneal haze.

**Conclusion:**

Low BW and older maternal age are the most important predictors of corneal haze in premature infants. Premature infants with corneal haze could carry more systemic and ocular morbidities. Hence they may require more clinical attention. Corneal haze is unlikely to hinder the treatment of ROP. However, it is possible that corneal haze could hinder the examination of ROP in some infants. If corneal haze does interfere with ROP screening, a closer, more conservative follow-up schedule with a senior ophthalmologist experienced in managing ROP is recommended.

## Introduction

Corneas provide the major refractive power of the eyes and their clarity is important for proper human vision and visual development. Corneal transparency is related to intact corneal epithelium, parallel-arrayed collagen in the corneal stroma and normal functioning corneal endothelium [1]. Development of the cornea starts at the fifth gestational week [2]. Ectoderm and neural crest cells are responsible for primitive cornea formation and a number of genes are involved [2–5]. Corneal development is not complete until term [6].

Infantile corneal opacity has been associated with many problems, including congenital glaucoma, birth trauma, congenital hereditary endothelial dystrophy, or Peters’ anomaly [7]. All these conditions will result either in significant sequelae (e.g., corneal scarring) or persistent corneal opacity, which would eventually require a corneal transplant. Premature infants have been noted to have transient corneal haze [8, 9]. The etiology of this kind of corneal haze is unknown and possible associated factors in premature infants have not been studied. It is also not known how this corneal haze severity relates to clinical outcomes in premature infants, and if this phenomenon is consistent across different ethnic groups [10]. Our experience was that some premature infants with corneal haze are more clinically critical or have more systemic problems. Hence, we planned to study the characteristics of premature infants with corneal haze during retinopathy of prematurity (ROP) screening in a tertiary referral hospital. Possible mechanisms are also discussed.

## Methods

We performed a retrospective study of 261 consecutive patients seen by an experienced pediatric ophthalmologist (YHL) between 2005 and 2010 in our hospital’s neonatal intensive care unit (NICU) for screening and follow up of ROP. All patients received the same standard of care therapy/follow-up. Part of this data set was used and published for a completely separate analysis on ROP screening criteria [11]. This retrospective medical record review study was conducted according to the principles expressed in the Declaration of Helsinki and was approved by the Institutional Review Board (IRB) of Kaohsiung Medical University Chung-Ho Memorial Hospital, Kaohsiung, Taiwan. The data were accessed by the patients’ medical number. The IRB waived the need for consent from the parents/guardians for this retrospective medical record review study.

### Screening procedures for ROP

An ophthalmic consultation was done for every premature baby born with a birth weight (BW) <1501 g, a gestational age at birth (GA) <31 weeks, or an unstable clinical course, as determined by NICU neonatologists [11]. The International Classification of Retinopathy of Prematurity was used for ROP staging [12–14], and the revised criteria for ROP screening and treatment were used to determine treatment course [15–17]. The examiner was not masked to birth weight and gestational age of the infants. Normal saline is used to prevent the drying of the cornea. However, if corneal haze hindered proper retinal evaluation, another examination was performed at least 1 week earlier than the recommended schedule.

### Grading of corneal haze

Corneal haze was subjectively graded according to the following definitions: grade 0 = clear; grade 1 = delayed haze (Corneal haze not noted immediately after opening the eye, but noted later in the examination); grade 2 = mild/moderate haze (Corneal haze noted immediately after opening the eye, but it did not hinder retinal examination); and grade 3 = severe haze (Corneal haze noted immediately after opening the eye and it interfered with retinal examination). Examples of grades 2 and 3 corneal haze are shown in the Supporting Information (S1 and S2 Figs).

### Central corneal thickness and intraocular pressure measurement

We speculated that intraocular pressure (IOP) and central corneal thickness (CCT) could be abnormal in infants with corneal haze. The reason why we measured these data was for differential diagnosis. IOP and CCT were measured at the time of ROP screening and during scheduled follow-up examinations. Before examination, the examination content and procedures regarding IOP and CCT measurements were explained to the parents/guardians, and oral consent was obtained. Before IOP and CCT measurement, a drop of proparacaine was instilled, and the eyelids were retracted with a speculum. Normal saline is used to prevent the drying of the cornea. Infants generally tolerated the speculum well, becoming calm and quiet within a few seconds with pacifier, soothing pats and caresses. Once infants were calmed, the ophthalmic examination of external and internal ocular structures continued. The IOP was measured with a Tono-Pen XL applanation tonometer (Reichert Inc., Depew, NY). The CCT was measured using a DGH 500 ultrasound pachymeter (DGH Technology Inc., Exton, PA). The measurement of IOP and CCT was discontinued once the cornea became clear or when the ROP follow-up was no longer required.

### Data collection

Gestational age (GA) and the postmenstrual age (PMA) were defined according to the accepted terminology [18]. The following parameters were examined from the medical records at the initial and follow-up examinations: gender, BW, GA, associated pregnancy and birth history, mother’s age, related systemic problems (packed red blood cells [RBC] transfusion, bronchopulmonary dysplasia [BPD], patent ductus arteriosus [PDA], intraventricular hemorrhage [IVH], respiratory distress syndrome [RDS], and hyperbilirubinemia), days on oxygen, status of retinopathy, corneal haze grade, CCT, and IOP.

### Data analysis

The first data analysis step consisted of analyzing demographic data and comparing them between premature infants with (corneal haze group) and without (control group) corneal haze. Corneal haze grading of the right eye was selected for characteristics analysis. The chi-square or Fisher’s exact test was used to compare differences in rates between the two groups, and the t-test was used to compare mean differences between the two groups. Our hypothesis is that the corneal haze grading, CCT, and IOP would change with PMA (i.e. age for process of maturation), hence the correlations between the three variables and PMA were analyzed. When analyzing how corneal haze grading, CCT, and IOP changed with PMA, only the data obtained in the initial examination were used. For correlation analysis, Pearson or Spearman test was used. Statistical significance was defined as a two-sided p value of <0.05. If significant differences were found in more than one demographic parameter, a multivariate logistic regression was used to determine the most significant predictors of transient corneal haze using Enter methods. The Hosmer-Lemeshow test was used to examine how well the logistic regression analysis model fit the data. SPSS statistical software (version 18.0, SPSS Inc., Chicago, IL) was used. Since we did not measure CCT in premature infants with no corneal haze, historical CCT data collected from premature infants were used for comparison [9]. The number of cases, mean, and standard deviation were used in t-tests [10, 19] using an online t-test calculator (http://www.graphpad.com/quickcalcs/ttest1.cfm). When comparing among study groups, the missing data were excluded in the analyses.

The second phase of data analysis was done if multiple significant demographic predictors of the condition were found in the first analysis. We investigated whether corneal haze severity (grades) was significantly affected by the same parameters using analysis of variance (ANOVA). Characteristics between right eyes and left eyes were also compared for interocular symmetry analysis [20]. Fisher’s least significant difference (LSD) or Dunnet C test was used for *post-hoc* comparisons; the Bonferroni correction was used for multiple comparisons.

## Results

### Demographic comparisons between premature infants with and without corneal haze

All the patients were Taiwanese. Of the 261 patients, thirty five infants (13.4%) were noted to have corneal haze. The BW and GA were significantly lower in the corneal haze group than in the control group (Table 1). For more detailed data distribution, please see Supporting information (S1, S2 and S3 Tables). The median and range of the BW of the infants without corneal haze were 1550g and 717 to 3250g and those with corneal haze were 1090g, and 541 to 1662g. The median and range of the GA of the infants without corneal haze were 31.6 weeks and 24.6 to 36.7 weeks, and those with corneal haze were 28.4 weeks and 24.3 to 38.1 weeks. Nine infants with corneal haze (25.7%) progressed to develop stage 3 ROP while 9.8% of the infants without corneal haze progressed to stage 3 ROP (p = 0.020). Five infants with corneal haze (14.3%) required laser treatment while 5.3% of the infants without corneal haze required laser treatment (p > 0.05). 74.3% of those with corneal haze and 90.2% of those without corneal haze had ROP less severe than stage 3. Three of the 93 (96.8%) infants who never developed ROP showed corneal haze.

**Table 1:** Comparison between eyes with and without corneal haze.

	Haze	No haze	p values	A Logistic Regression Model	
	mean (SD)	mean (SD)		Adjusted OR	Adjusted p value
Number	35	226	-	-	-
GA (week)	28.7 (2.9)	31.2 (2.6)	<0.001*	0.970 (0.715–1.316)	0.846
BW (g)	1087.1 (301.1)	1522.6 (422.9)	<0.001*	0.998 (0.996–1.000)	0.024*
SGA			0.259		
Yes	9	40		-	-
No	26	186			
Sex			0.655		
Male	20	120		-	-
Female	15	106			
PDA			0.221		
Yes	21	106		-	-
No	14	111			
RDS			0.080		
Yes	32	171		-	-
No	3	46			
BPD					
Yes	10	32	0.026*	0.531 (0.113–2.509)	0.425
No	21	171		Reference	
IVH			0.825		
Yes	7	40		-	-
No	28	177			
Hyperbilirubinemia			0.116		
Yes	33	182		-	-
No	2	34			
Transfusion			<0.001*		
Yes	27	96		0.927 (0.270–3.179)	0.927
No	8	120		Reference	
Days on oxygen	52.7 (50.4)	18.9 (23.9)	0.001*	1.017 (0.993–1.043)	0.171
Laser			0.060		
Yes	5	12		-	-
No	30	214			
Stage 3 ROP			0.020*		
Yes	9	22		1.117 (0.340–3.675)	0.855
No	26	202		Reference	
Mother’s age (year)	33.1 (5.0)	30.4 (4.9)	0.004*	1.104 (1.001–1.217)	0.049*

BPD = bronchopulmonary dysplasia; BW = birth body weight, GA = gestational age at birth, IVH = intraventricular hemorrhage, OR = odds ratio, PDA = patent ductus arteriosus, RDS = respiratory distress syndrome, ROP = retinopathy of prematurity, SD = standard deviation, SGA = small for gestational age

*indicates p < 0.05

Infants with corneal haze were more likely to have packed RBC transfusion and to have longer days on supplemental oxygen (Table 1). Additionally, the mother’s age at the time of delivery was significantly older in the corneal haze group (Table 1). Logistic regression analysis revealed that BW and mother’s age were the most significant factors associated with corneal haze (Table 1).

### Corneal haze, CCT, and IOP

**Fig 1:**
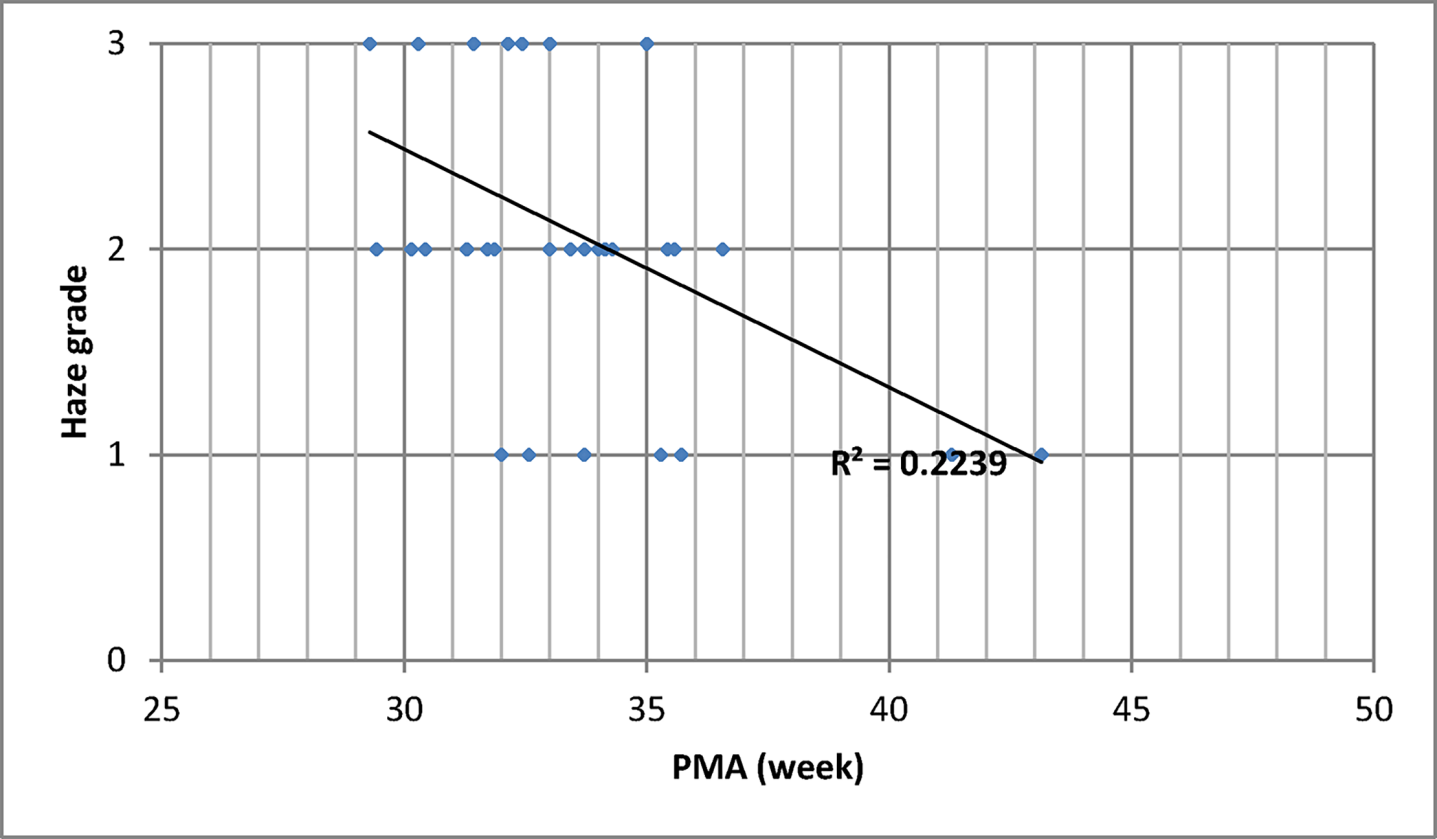
Corneal haze grades and post-menstrual age. Grade 0 = clear; Grade 1 = delayed haze (Corneal haze not noted immediately after opening the eye, but noted later in the examination); Grade 2 = mild/moderate haze (Corneal haze noted immediately after opening the eye, but it did not hinder retinal examination); Grade 3 = severe haze (Corneal haze noted immediately after opening the eye and it interfered with retinal examination). PMA: postmenstrual age. *The severity of corneal haze decreased with infants’ PMA (p = 0.014).

When comparing the ophthalmic findings in infants with different corneal haze grades, those of the grade 1 group were similar to those of the grade 0 group, but not to the grade 2 or 3 groups (Table 2). Thirty-seven percent of those of GA 26 weeks or fewer were noted to have corneal haze. The severity of corneal haze decreased with infants’ PMA (p = 0.014, Fig 1). The average PMAs of infants who presented with grade 0, 1, 2 and 3 corneal haze were 37.3, 36.3, 34.2 and 33.2 weeks, respectively. For the 35 infants, the corneal haze was assessed 5 times in 10 infants, 4 times in 1 infants, 3 times in 8 infants, twice in 3 infants and only once in 13 infants. A gradually decrease in corneal haze was noted when the infants were more mature. When corneal haze diminished completely, the average age was 12.0 ± 3.0 weeks and the PMA was 40.1 ± 2.9 weeks. All the corneal haze diminished completely by 46.6 weeks of PMA.

**Table 2:** Comparison between different grades of corneal haze.

	Grade 0	Grade 1	Grade 2	Grade 3
	mean (SD)	mean (SD)	mean (SD)	mean (SD)
Number	226	7	18	10
GA (weeks)	31.2 (2.6)	31.8 (3.6)	28.1 (2.3)*	27.8 (2.1)*
BW (g)	1522.6 (422.9)	1372.0 (241.5)	1007.3 (268.8)*	1031.3 (296.2)*
Days on oxygen	18.9 (23.9)	18.0 (21.2)	48.4 (37.4)	76.8 (67.8)
Mother’s age (years)	30.4 (4.9)	33.6 (4.4)	33.4 (3.1)*	32.1 (7.7)

SD: standard deviation; GA: gestational age at birth; BW: birth body weight. Post-hoc by Bonferroni test (GA, and BW) or Dunnett C test days on oxygen, and mother’s age

*p < 0.017 (statistically significant after Bonferroni correction), compared with those without haze

**Fig 2:**
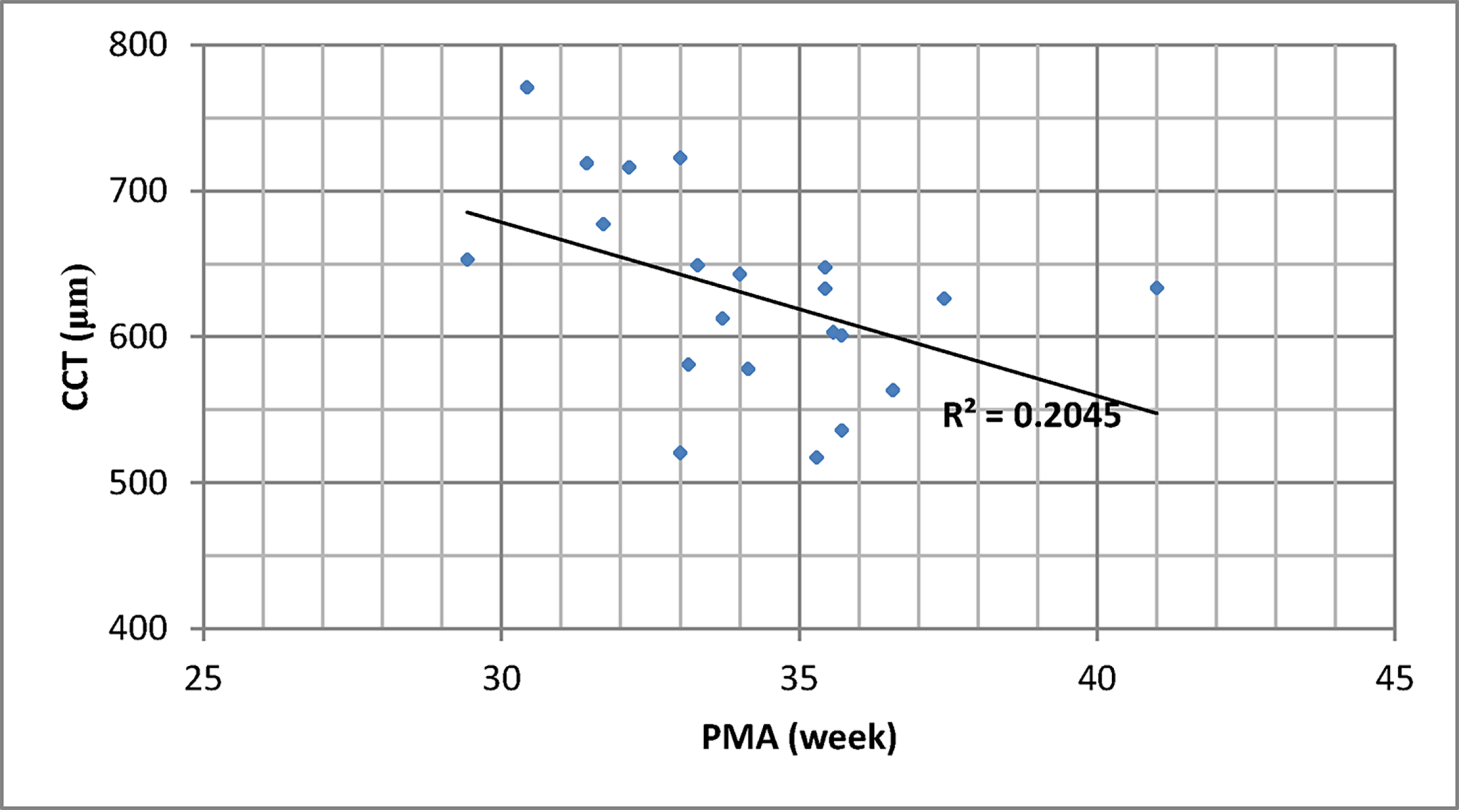
Central corneal thickness and post-menstrual age. CCT: central corneal thickness. PMA: post-menstrual age. *CCT decreased with PMA (p = 0.040).

Average CCT was 618.0 (± 66.6) μm. CCT decreased with PMA (p = 0.040, Fig 2). Our CCT measurements (infants with corneal haze) were not significantly different from historical CCT data collected from premature infants (Table 3) [9]. CCT correlated with corneal haze (p = 0.011). The mean CCTs were 577.1, 587.7, 629.0, and 658.0μm for grade 0, 1, 2, and 3 corneal haze, respectively.

**Table 3:** Central corneal thickness measurement comparisons.

	CCT in hazy cornea (present study)		Normal CCT in Kirwan’s study		
PMA (weeks)	CCT (μm) (SD)	n (eyes)	CCT (μm) (SD)	n (eyes)	p value*
<33	707.3 (45.1)	5	691 (87)	35	0.686
33–35	614.4 (59.9)	16	648 (72)	35	0.111
36–38	602.5 (42.3)	6	605 (59)	35	0.922
≥39	559.0 (44.3)	5	564 (34)	35	0.768

CCT = central corneal thickness, PMA = post-menstruation age, SD = standard deviation, n = number of cases

*Calculated at http://www.graphpad.com/quickcalcs/ttest2.cfm (date: Jan. 31, 2017)

**Fig 3:**
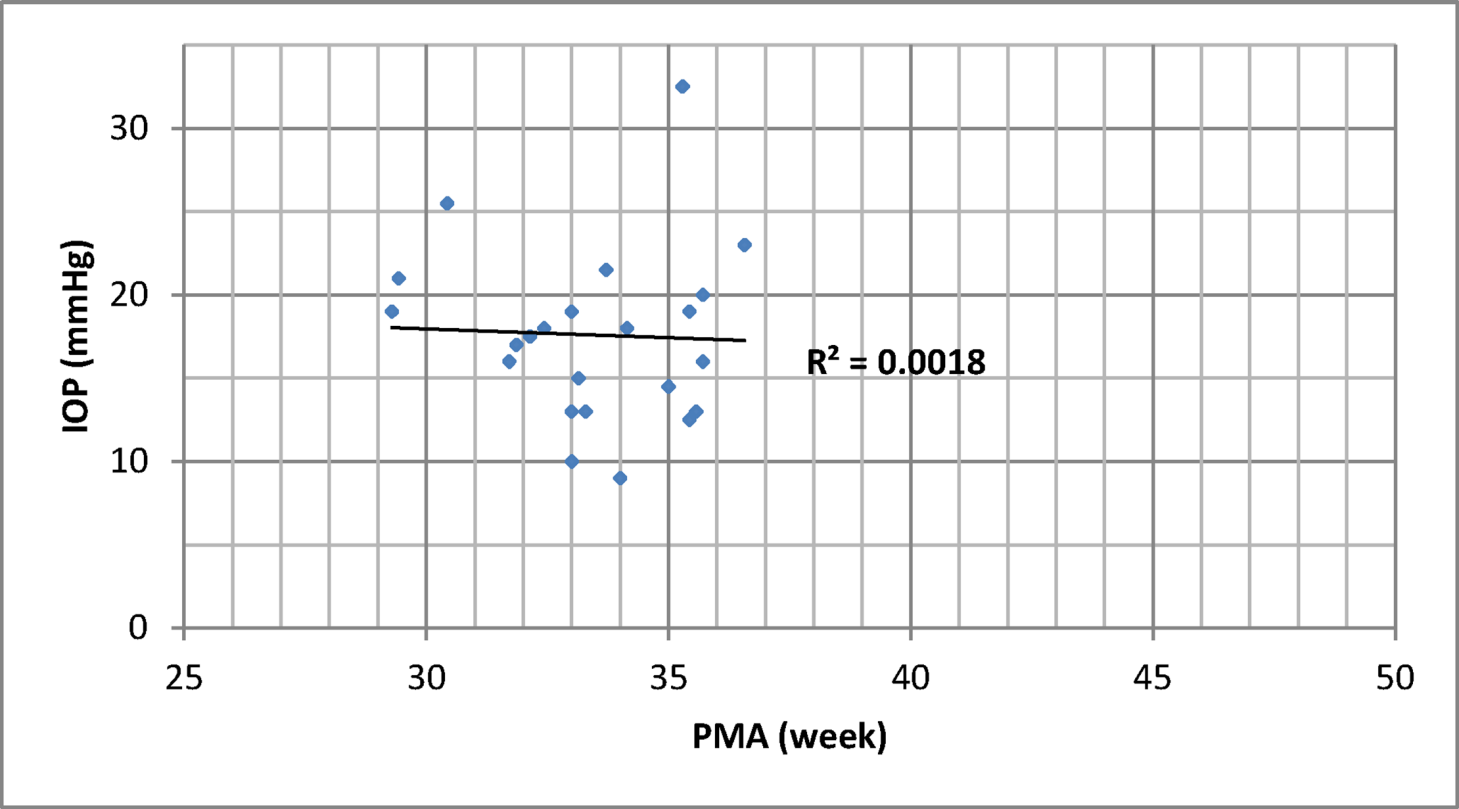
Intraocular pressure and post-menstrual age. IOP: intraocular pressure. PMA: post-menstrual age. IOP did not vary with PMA (p > 0.05).

Average IOP was 17.5 (± 5.2) mmHg and IOP did not vary with PMA or corneal haze (p > 0.05, Fig 3).

### Interocular symmetry

When ophthalmic findings were compared between eyes in the corneal haze group, no significant differences were found, including C/D ratio, with or without laser treatment, corneal haze staging, and ROP severity. There was no statistically significant difference regarding cup to disc ratio versus corneal haze grade (p > 0.05).

## Discussion

This study showed that the severity of corneal haze decreased with age and achieved its transparency at an average age of 40 weeks PMA. In addition, more than one tenth of the premature infants were noted to have corneal haze. Our study revealed that BW is one of the most important predictors of corneal haze. Lower birth weight has been associated with increased rates of several systemic morbidities, i.e., coronary heart disease, metabolic syndrome, and osteoporosis [21]. It has been proposed that early-life experiences could produce long-term functional and structural changes. The concept of developmental plasticity has also been introduced to explain the possible mechanism. Preterm birth has been associated with myopia and astigmatism [22, 23]. Moreover, Ecsedy et al. revealed that children with a history of preterm birth tend to have lower- and higher-order aberrations in their corneas [24]. If corneal haze is associated with long-term ocular morbidity requires further study.

It is well known that extreme maternal age (i.e., teenager, >35 years) is considered a risk in pregnancy [25, 26]. Additionally, optic nerve hypoplasia and anisometropia are associated with young maternal age and old maternal age, respectively [27, 28]. The current study adds corneal haze as another risk of old maternal age.

As expected, the corneal haze decreased with PMA. The cornea is derived from surface ectoderm and the neural crest cells. Although corneal structures are grossly established when the eyelids are separated at the fifth month of gestation [6], the corneal maturation process is not complete until term [6]. An intact corneal epithelium, parallel-arrayed corneal stromal collagen, and normal corneal endothelium are all required for corneal transparency [1]. Transparency is also decreased in an edematous cornea. We speculated that corneal haze could be associated with changes in CCT or IOP. Our study showed that the CCT of premature infants with corneal haze was not different from those without, and that IOP has no role in corneal haze. Despite our results showing CCT correlated with corneal haze grading, this could just reflect that both the CCT and corneal haze decrease as the infants mature.

Corneal haze in premature infants is probably heterogeneous. A small portion of patients had delayed corneal haze (grade 1). Although the exact retinal examination time was not recorded, those with delayed corneal haze were not more immature or presenting more severe ROP, hence this group was unlikely to need more time to examine their retinas. Additionally, normal saline was routinely used, when necessary, to prevent the cornea over-drying, it is unlikely that this type of corneal haze is due to prolonged exposure. Transient corneal opacity has been noted in patients with Raynaud’s disease during exposure to cold temperatures [29]. The majority of the body of our patients was in an incubator and covered by clothes, but the infants’ heads were briefly exposed to lower temperatures during retinal examination. It is possible that some of the more sensitive premature neonates responded to the temperature change (from 30–32°C to 24–26°C). The corneal opacity in our grade 1 patients may possibly be attributed to a similar phenomenon. Additionally, the characteristics of patients with grade 1 corneal haze were different from those with grades 2 or 3. We believe that grade 1 corneal haze etiology and mechanism is different from that of grade 2 and grade 3. A slight NICU room temperature increase or a specially-prepared examining room for these infants may minimize corneal haze during retinal examination.

Generally, poor outcome indicators of ROP were observed after 31 weeks PMA [30], and more severe ROP developed around 35–38 weeks PMA [31]. In the current study, despite corneal haze being associated with stage 3 ROP and usually lasting until 40 weeks PMA, higher grades of corneal haze had mostly disappeared by 33–34 weeks PMA. Hence corneal haze is unlikely to hinder the treatment of ROP. Contrarily, it is possible that corneal haze could hinder the examination of ROP in some infants. If corneal haze does interfere with ROP screening, a closer, more conservative follow-up schedule with a senior ophthalmologist experienced in managing ROP should be implemented. With modern imaging equipment, such as the RetCam Digital Retinal Camera (Massie Research Laboratories Inc., Pleasanton, CA), physicians can sometimes observe fundus details more easily than with indirect ophthalmoscopy.

It is interesting that chronic obstructive pulmonary disease has been associated with a lower endothelial cell density (ECD), a lower percentage of hexagonal cells and a higher variance of cell size [32, 33]. Similar findings were also observed in sickle cell anemia patients. All these factors are poor indicators of corneal transparency. Our premature infants with BPD and those who received pRBC transfusion were also more likely to have corneal haze. Whether the two conditions share some common pathogeneses requires further study.

Our results confirmed our speculation that some of the infants with corneal haze were clinically unstable. Hence they may require more clinical attention. The current study could also remind the developing NICUs to enhance the care of their patients. There are some limitations in this study: The IOP was measured while the infants were awake, which may lead to overestimate the real IOP in some infants. The retrospective nature of this study may have led to an underestimation of less severe corneal haze. Additionally, this preliminary study could not assess the reproducibility of this grading scale and the exact examination time of grade 1 corneal haze. Further study is also needed to assess the reproducibility of this new grading system. In contrast to Dr. McCormick’s article stating that the cornea of a fetus at 26 weeks’ gestation is almost intransparent [8], less than half of the infants in the present study at age of 26 weeks or fewer had corneal haze. If this discrepancy is due to the ethnic difference requires further study. Future prospective studies using more advanced imaging techniques, including anterior segment optical coherence tomography or corneal interferometry, may be helpful.

## Supporting information

S1 Fig An example of grade 2 corneal haze. (JPG)

S2 Fig An example of grade 3 corneal haze. (JPG)

S1 Table Clinical characteristics of premature infants by birth weight. (DOCX)

S2 Table Clinical characteristics of premature infants by gestational age. (DOCX)

S3 Table Clinical characteristics of premature infants by corneal haze grades. Interested researchers could access the full data if their study is approved by the Institutional Review Board of Kaohsiung Medical University Chung-Ho Memorial Hospital, Kaohsiung, Taiwan.
(DOCX)

S1 File STROBE_checklist_for cross-sectional study. (DOCX)
