# Supplementary figures and images for: The characteristics of premature infants with transient corneal haze
Source: PLoS One. 2018 Mar 29;13(3):e0195300. doi: 10.1371/journal.pone.0195300 (PMC5875869; doi:10.1371/journal.pone.0195300)

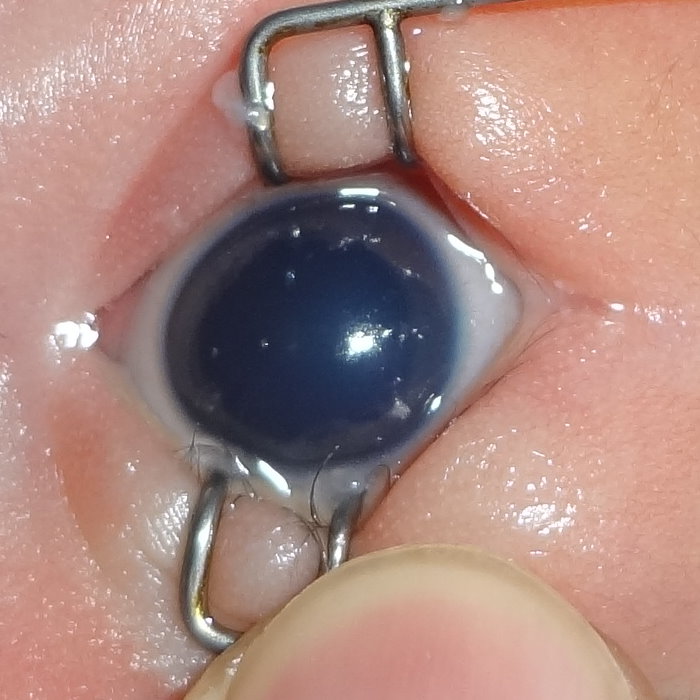

Supplement: S1 Fig — (JPG) [file pone.0195300.s001.JPG]

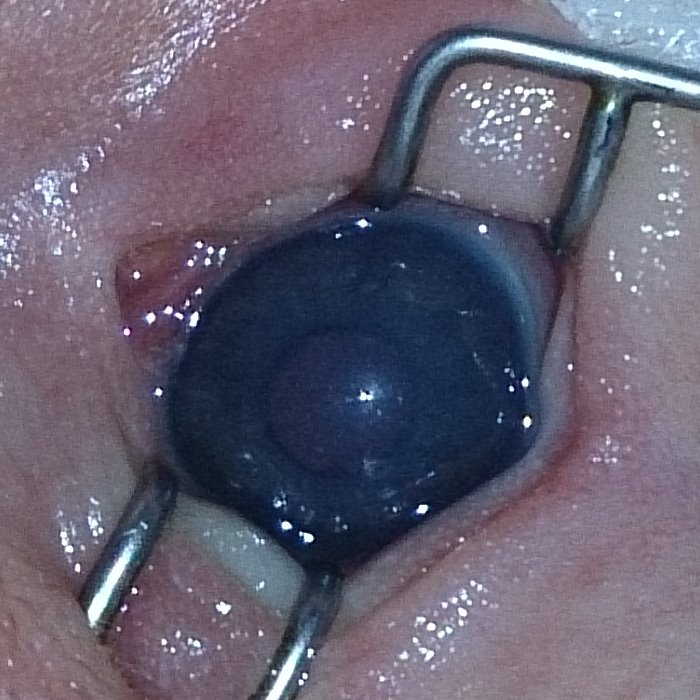

Supplement: S2 Fig — (JPG) [file pone.0195300.s002.JPG]
