# Supplementary material for: The characteristics of premature infants with transient corneal haze
Source: PLoS One. 2018 Mar 29;13(3):e0195300. doi: 10.1371/journal.pone.0195300 (PMC5875869; doi:10.1371/journal.pone.0195300)
Supplement: S1 Table — (DOCX) [file pone.0195300.s003.docx]

S1 Table. Clinical characteristics of premature infants by birth weight

| BW (g) | N (%) | Haze (%) | Male/Female | SGA | PDA | RDS | BPD | IVH | Hyperbilirubinemia | Transfusion | Days on O2  Mean (SD) | Laser | Stage 3 ROP | Mother’s age  Mean (SD) |
| --- | --- | --- | --- | --- | --- | --- | --- | --- | --- | --- | --- | --- | --- | --- |
| 501-1000 | 41 (15.7) | 15 (42.9) | 22/19 | 14 | 26 | 38 | 17 | 14 | 34 | 33 | 66.8 (47.6) | 12 | 17 | 31.2 (5.1) |
| 1001-1500 | 98 (37.5) | 17 (48.6) | 55/43 | 21 | 59 | 83 | 12 | 18 | 84 | 67 | 23.6 (21.1) | 5 | 13 | 30.8 (4.4) |
| 1501-2000 | 98 (37.5) | 3 (8.6) | 48/50 | 13 | 37 | 68 | 10 | 13 | 79 | 22 | 10.1 (14.2) | 0 | 1 | 30.5 (5.4) |
| 2001-2500 | 21 (8.0) | 0 | 12/9 | 1 | 5 | 13 | 1 | 1 | 17 | 1 | 7.5 (10.3) | 0 | 0 | 31.6 (5.5) |
| 2501-3000 | 1 (0.4) | 0 | 1/0 | 0 | 0 | 0 | 0 | 0 | 0 | 0 | - | 0 | 0 | - |
| >3000 | 2 (0.8) | 0 | 2/0 | 0 | 0 | 1 | 2 | 1 | 1 | 0 | 7.5 (0.7) | 0 | 0 | 27.5 (2.1) |
| Total | 261 (100) | 35 (100) | 140/121 | 49 | 127 | 203 | 42 | 47 | 215 | 123 | 23.3 (30.9) | 17 | 31 | 30.8 (5.0) |

BPD = bronchopulmonary dysplasia; BW = birth body weight, IVH = intraventricular hemorrhage, N = case number; PDA = patent ductus arteriosus, RDS = respiratory distress syndrome, Retinopathy of prematurity, SD = standard deviation, SGA = small for gestational age
