# Supplementary material for: The characteristics of premature infants with transient corneal haze
Source: PLoS One. 2018 Mar 29;13(3):e0195300. doi: 10.1371/journal.pone.0195300 (PMC5875869; doi:10.1371/journal.pone.0195300)
Supplement: S2 Table — (DOCX) [file pone.0195300.s004.docx]

S2 Table. Clinical characteristics of premature infants by gestational age

| GA (week) | N (%) | Haze (%) | Male/Female | SGA | PDA | RDS | BPD | IVH | Hyperbilirubinemia | Transfusion | Days on O2  Mean (SD) | Laser | Stage 3 ROP | Mother’s age  Mean (SD) |
| --- | --- | --- | --- | --- | --- | --- | --- | --- | --- | --- | --- | --- | --- | --- |
| 24-26.9 | 27 (10.3) | 10 (28.6) | 19/8 | 2 | 18 | 26 | 10 | 8 | 23 | 23 | 72.0 (42.2) | 11 | 15 | 31.0 (4.8) |
| 27-29.9 | 69 (26.4) | 15 (42.9) | 39/30 | 10 | 41 | 31 | 14 | 20 | 55 | 51 | 39.3 (34.2) | 6 | 12 | 29.9 (5.4) |
| 30-32.9 | 105 (40.2) | 8 (22.9) | 57/48 | 14 | 49 | 85 | 5 | 15 | 89 | 41 | 11.2 (10.1) | 0 | 2 | 31.1 (4.6) |
| 33-35.9 | 58 (22.2) | 1 (2.9) | 24/34 | 22 | 19 | 31 | 12 | 4 | 47 | 7 | 8.3 (16.2) | 0 | 2 | 31.1 (5.1) |
| >=36 | 2 (0.8) | 1 (2.9) | 1/1 | 1 | 0 | 0 | 1 | 0 | 1 | 1 | 0 | 0 | 0 | 33.5 (6.4) |
| Total | 261 (100) | 35 (100) | 140/121 | 49 | 127 | 203 | 42 | 47 | 215 | 123 | 23.3 (30.9) | 17 | 31 | 30.8 (5.0) |

BPD = bronchopulmonary dysplasia; GA = gestational age at birth, IVH = intraventricular hemorrhage, N = case number; PDA = patent ductus arteriosus, RDS = respiratory distress syndrome, ROP = retinopathy of prematurity, SD = standard deviation, SGA = small for gestational age
