# Supplementary material for: The characteristics of premature infants with transient corneal haze
Source: PLoS One. 2018 Mar 29;13(3):e0195300. doi: 10.1371/journal.pone.0195300 (PMC5875869; doi:10.1371/journal.pone.0195300)
Supplement: S3 Table — Interested researchers could access the full data if their study is approved by the Institutional Review Board of Kaohsiung Medical University Chung-Ho Memorial Hospital, Kaohsiung, Taiwan. (DOCX) [file pone.0195300.s005.docx]

S3 Table. Clinical characteristics of premature infants by corneal haze grades

| Grade | N (%) | GA (week)  Mean (SD) | BW (g)  Mean (SD) | Male/Female | SGA | PDA | RDS | BPD | IVH | Hyperbilirubinemia | Transfusion | Days on O2  Mean (SD) | Laser | Stage 3 ROP | Mother’s age  Mean (SD) |
| --- | --- | --- | --- | --- | --- | --- | --- | --- | --- | --- | --- | --- | --- | --- | --- |
| 0 | 226 (86.6) | 31.2 (2.6) | 1522.6 (422.9) | 120/106 | 40 | 106 | 171 | 32 | 40 | 182 | 96 | 18.9 (23.9) | 12 | 22 | 30.4 (4.9) |
| 1 | 7 (2.7) | 31.8 (3.6) | 1372.0 (241.5) | 4/3 | 3 | 3 | 6 | 0 | 1 | 7 | 4 | 18.0 (21.2) | 0 | 2 | 33.6 (4.4) |
| 2 | 18 (6.9) | 28.1 (2.3) | 1007.3 (268.8) | 9/9 | 6 | 9 | 17 | 5 | 3 | 17 | 13 | 48.4 (37.4) | 3 | 4 | 33.4 (3.1) |
| 3 | 10 (3.8) | 27.8 (2.1) | 1031.3 (296.2) | 7/3 | 1 | 9 | 9 | 5 | 3 | 9 | 10 | 76.8 (67.8) | 2 | 3 | 32.1 (7.7) |
| Total | 261 (100) | 30.8 (2.7) | 1464.2 (434.4) | 140/121 | 49 | 127 | 203 | 42 | 47 | 215 | 123 | 23.3 (30.9) | 17 | 31 | 30.8 (5.0) |

BPD = bronchopulmonary dysplasia; BW = birth body weight, GA = gestational age at birth, IVH = intraventricular hemorrhage, N = case number; PDA = patent ductus arteriosus, RDS = respiratory distress syndrome, ROP = retinopathy of prematurity, SD = standard deviation, SGA = small for gestational age
